# Influence of Microalgal Cell Division Tendency on OD to DCW Conversion Factor and Chlorophyll Contents

**DOI:** 10.4014/jmb.2412.12049

**Published:** 2025-04-09

**Authors:** Hyeong Woon Choe, DanA Kim, Young Jun Choi, Hyun Gi Koh, Won-Kun Park

**Affiliations:** 1 Department of Chemistry, Sangmyung University, Seoul 03016, Republic of Korea; 2 Department of Biological Engineering, Konkuk University, Seoul 05029, Republic of Korea; 3 Advanced Materials Program, Konkuk University, Seoul 05029, Republic of Korea; 4 Department of Biological & Chemical Engineering, Hongik University, Sejong-si 30016, Republic of Korea

**Keywords:** Microalgae, cell division, growth measurement, cell size, conversion factor

## Abstract

As the importance of achieving a carbon-neutral society grow, research on the production of microalgal bioproducts have gained significant attention. Among the production processes, cultivation has emerged as a critical step as it determines overall productivity and feasibility. However, changes in cell properties during cultivation pose challenges, and there is currently no direct method to simultaneously assess cell growth, cellular division properties and cell enlargement. In this study, two green algae (*Chlorella* sp. ABC001 and *Chlorella* sp. HS2) were cultivated under photo and mixotrophic conditions to evaluate their growth, cell size profile, and changes in optical density. The mixotrophic condition resulted in 2.1 and 1.6-fold higher biomass yields than the photoautotrophic condition for ABC001 and HS2, respectively at day 4. Over the 4-day cultivation, cell sizes ranged from 2.51 to 5.57 μm for ABC001 and from 2.56 to 4.41 μm for HS2. By analyzing changes in the conversion factor between dry cell weight (DCW) and optical density (OD), it was observed that variations in the slope correlated with changes in cell size. Additionally, chlorophyll content fluctuated during cultivation, reaching maximum levels (14.34 and 16.58 μg/mg biomass, respectively) under phototrophic condition on day 2. This study highlights the relationship between cell division tendencies, DCW, cell size, and OD, demonstrating their critical role in determining cellular component content. For future optimization of algal product production processes, further research on these cellular differentiation mechanisms will be essential.

## Introduction

As microalgal biofuel gain recognition as renewable and carbon neutral energy sources, algal processes have been extensively studied and enhanced [1]. Among these, the cultivation process has received particular attention, as it determines the overall productivity of the entire process and constitutes the largest portion of production costs [2, 3]. The productivity of target products can be significantly influenced by the culture conditions and strategies employed. For example, nitrogen limited culture condition enhanced the lipid content [4, 5], while sulfur-depleted conditions increase hydrogen production yield [6]. Thus, proper culture conditions and effective cell monitoring systems are essential to optimize cultivation.

In this circumstance, various cell growth monitoring methods have been suggested and utilized in the cultivation process to achieve as much biomass as possible [7]. One approach involves counting cells using a microscope [8, 9]. This method requires only a small culture volume (up to 20 ml) and provides a direct, straightforward measurement. However, it often necessitates multiple dilution steps to achieve a countable concentration range, making the process time-consuming and prone to error [10]. While cell counting is reliable for assessing cell numbers and observing cell division, it does not account for changes in cell size, volume, or weight.

On the other hand, dry cell weight (DCW) is another effective method for determining cell mass [11, 12]. This technique requires a specific culture volume, and it takes at least several hours to be measured because of harvesting and drying process. The required volume could be varied from 1 to 50 ml depend on the cell concentration and the time for process is deferred from 4 to 12 h by the culture volume to be dried and drying methods. Although DCW measurement is the most reliable mass-based method, it does not allow immediate assessment of cell growth.

For the purpose of fast and easy measurement, optical density (OD) could be used in the form of indirect measuring method [12, 13]. OD measures the light absorbance of suspended cells in a culture and can be adjusted to specific wavelengths to targeted particular pigments or cells (625 nm for blue pigment *e.g.* phycocyanin, 650 nm for green algae, 680 nm for green pigment *e.g.* chlorophyll, and 750 nm for particles in the form of turbidity which is widely used for various types of microalgae) [14, 15]. The primary advantages of this method include fast measurement (less than 30 sec) and small culture volume required for the measurement. It usually takes about 1 ml and could be smaller than that when it needs dilution. However, for the measurement of cell growth, it should be correlated with cell number or DCW to be compared as a growth parameter [16]. The correlation between OD and cell number or DCW measured once and OD value is used to figure out estimated DCW or cell number with the calculation using the correlation. However, this estimation is sometimes not matched well with actual values of cell number or dry cell weight. 

Reliable conversion between OD and DCW (or cell number) which means proportional relationship between two factors, requires consistent cell size and shape throughout the cultivation period [15, 17, 18]. While cell count and DCW are direct measurements, OD calculates growth based on light absorbance, which can vary with changes in cell morphology. That means different cell size or shape makes different tendency of light absorption. This limitation has led several studies to emphasize that OD-based correlations are valid only when cell size and shape remain constant [17, 18].

Microalgae, however, often exhibit morphological changes influenced by culture conditions. For example, algal cells follow a circadian rhythm that regulates their cell division during light/dark cycles to maximize productivity [19]. These cycles create distinct interdivision and division periods [20], during which cell size and shape may vary. *Chlamydomonas*, for instance, is oval-shaped with flagella under normal conditions but becomes circular and enlarged during the interdivision period when the flagella are removed [21]. After cell division, daughter cells are smaller than normal cells [21]. Such variations can introduce errors when OD is used with conversion equations to calculate DCW or cell number, potentially leading to significant inaccuracies in estimating cell density during cultivation. Given the widespread use of OD-to-DCW conversion in the actual algal biomass production process, such errors could substantially impact process efficiency.

Thus, in this study, we investigated cell size differentiation during the cultivation under two different culture conditions (photoautotroph and mixotroph) with two different green algae (*Chlorella* sp. ABC001 and *Chlorella* sp. HS2). Cell growth was measured via both DCW and OD at 750 nm, and changes in the conversion ratio between DCW and OD_750 nm_ were analyzed in relation to cell size variations during division. Additionally, chlorophyll content, a major component of algal biomass, was examined to explore the relationship between cell division properties and cellular composition. This study provides valuable insights into cell division dynamics, their impact on algal productivity, and improved approaches for measuring cell growth.

## Materials and Methods

### Strain and Maintenance Conditions

*Chlorella* sp. ABC001 was provided by Advanced Biomass R&D Center, Deajeon, Korea and , *Chlorella* sp. HS2 was kindly provided by Dr. Hee-Sik Kim in Korea Research Institute of Bioscience and Biotechnology (KRIBB). Both algal strains were maintained on BG11 agar plates (15 g bacto agar, 1.5 g NaNO_3_, 0.04 g K_2_HPO_4_·2H_2_O, 0.075 g MgSO_4_·7H_2_O, 0.036 g CaCl_2_·2H_2_O, 0.006 g citric acid, 0.02 g Na_2_CO_3_, 0.006 g ferric ammonium citrate, 0.001 g Na-EDTA, and 1 ml A5 trace element solution in 1 L DI water) at 35°C in the incubator supplied with 5%CO_2_ and illuminated by five 55 W fluorescence lamps. The A5 Trace element was prepared by dissolving 2.86 g H_3_BO_3_, 1.81 g MnCl_2_·4H_2_O, 0.22 g ZnSO_4_·7H_2_O, 0.39 g NaMoO_4_·2H_2_O, 0.079 g CuSO_4_·5H_2_O in 1 L DI water.

### Culture Conditions

All the experiments were conducted with activated cells to minimize the lag phase caused by the absence of adaptation between cell maintenance conditions and the actual cultivation conditions. Cells maintained on agar plates were activated for one week in 200 ml cell culture flasks with a 100 ml working volume. Activation was performed at 35°C under five 55 W fluorescent lamps and 120 rpm for 24 h. BG11 medium was used for photoautotrophic (phototrophic) condition and 5 g/l glucose was added to the BG11 medium for mixotrophic conditions. After the activation process, cells were harvested with centrifugation at 3,000 rpm for 3 min and washed twice with fresh BG11 medium. The cell inoculum was prepared at 5 × 10^6^ cells/ml for a 200 ml working volume in 250 ml baffled flaks. BG11 medium and BG11 medium supplemented with 5 g/l glucose were used for phototrophic and mixotrophic conditions, respectively. A 5% CO_2_ was directly introduced into the baffled flask with 1 vvm. The temperature was maintained at 35°C with illumination provided by five 55 W fluorescence lamps for 24 h, and the rotation speed was set to 140 rpm. For the sampling, 10 ml cultures were pulled out from the flasks and total less than 50 ml cultures were used. All the experiments were repeated at least three times.

### Analysis Methods

**Cell count & cell size.** Cell size was measured daily using Cellometer Vision (Nexcelom, USA). A 20 ml culture sample was loaded into counting chamber, and image were captured by Cellometer. Cell size was determined based on image analysis [22]. The device operates using a cytometric analysis mechanism, minimizing the risk of inaccurate cell size estimation, even under compressed conditions with a small volume on the glass slide. All the measurements were performed at least six times, with measurement counting a minimum of 2,000 cells. The average value of these measurements was used for analysis.

**Optical density.** Cell growth measurement based on optical density was conducted every day using UV/Visible Spectrophotometer (Hach, DR2800, USA) at 750 nm, which is used for particle-based measurement. All the samples were thoroughly mixed using a vortex mixer (Vortex-Genie 2, USA) before measurement. For high-density samples (OD_750 nm_ > 0.9), a 10-fold dilution with the same medium was applied to prevent underestimation of cell density. All the measurements were performed at least twice, and the average value was used for analysis.

**Dry cell weight.** Cell growth was measured daily based on dry cell weight. A 10 ml sample was filtered through a 1.2 mm glass filter (GF/C 45 mm, Waters) and dried overnight in a 100°C drying oven. The dry cell weight per liter medium was calculated using Eq. (1).

$$\text{Dry cell weight per L }\left(\text{g/l}\right)=\frac{m_{2}-m_{1}}{a}$$ (1)

*m*_2_: weight of dried biomass + filter (mg), *m*_1_: weight of pre-dried filter (mg), and *a*: filtered culture volume (ml)

**Chlorophyll analysis.** To determine the chlorophyll content in the cell, 1 ml of culture was centrifuged at 3,000 rpm for 5 min and the supernatant was removed. Afterward, 5 ml of methanol was added, and the sample was stored at 4°C in the refrigerator, covered with aluminum foil, for a week. Most samples exhibited decolorized cell at the bottom of tubes, however, some samples retained green-colored cells after the extraction. These samples were disrupted using bead beater (Biospec Mini Beadbeater, USA) using zirconium bead (0.5 mm, Biospec, USA) for 30 sec with an icing step. After the disruption, the sample was centrifuged again, and the supernatant was measured at 652 and 665 nm using a UV/Vis Spectrophotometer. The chlorophyll a and the chlorophyll b concentrations were calculated using Eq. (2), suggested by Ritchie (2006) [23].

$$\begin{array}{l} \text{Chl }a\left(\mu g/ml\right)=-8.0962\times OD_{652}+16.5169\times OD_{665}\\ \text{Chl }b\left(\mu g/ml\right)=27.4405\times OD_{652}-12.1688\times OD_{665} \end{array}$$ (2)

**Glucose analysis.** Glucose analysis was performed using the anthrone method [4]. The anthrone solution was prepared by dissolving 2 g of anthrone in 100 ml of 72 wt% H_2_SO_4_. For the analysis, the samples were centrifuged at 13,000 rpm for 1 min, and 0.1 ml of the supernatant was diluted 10-folds. Then, 0.15 ml of diluted sample was transferred into 1.5 ml Eppendorf tube with a safety cap and 0.75 ml of the anthrone solution was added. The samples were then incubated in boiling water for 10 minutes and subsequently cooled to room temperature. The cooled samples were measured at 625 nm using UV/Vis Spectrophotometer, and glucose concentration was calculated using a standard curve (Eq. (3)) obtained with glucose standards (0, 50, 100, 200, 500, and 1,000 mg/ml).

$$\text{Glucose concentration (g/l)}=1.5993\times OD_{625}-0.021282\left(R^{2}=0.9969\right)$$ (3)

## Results and Discussion

### Growth Differences between Photoautotrophic and Mixotrophic Conditions

**Fig. 1:**
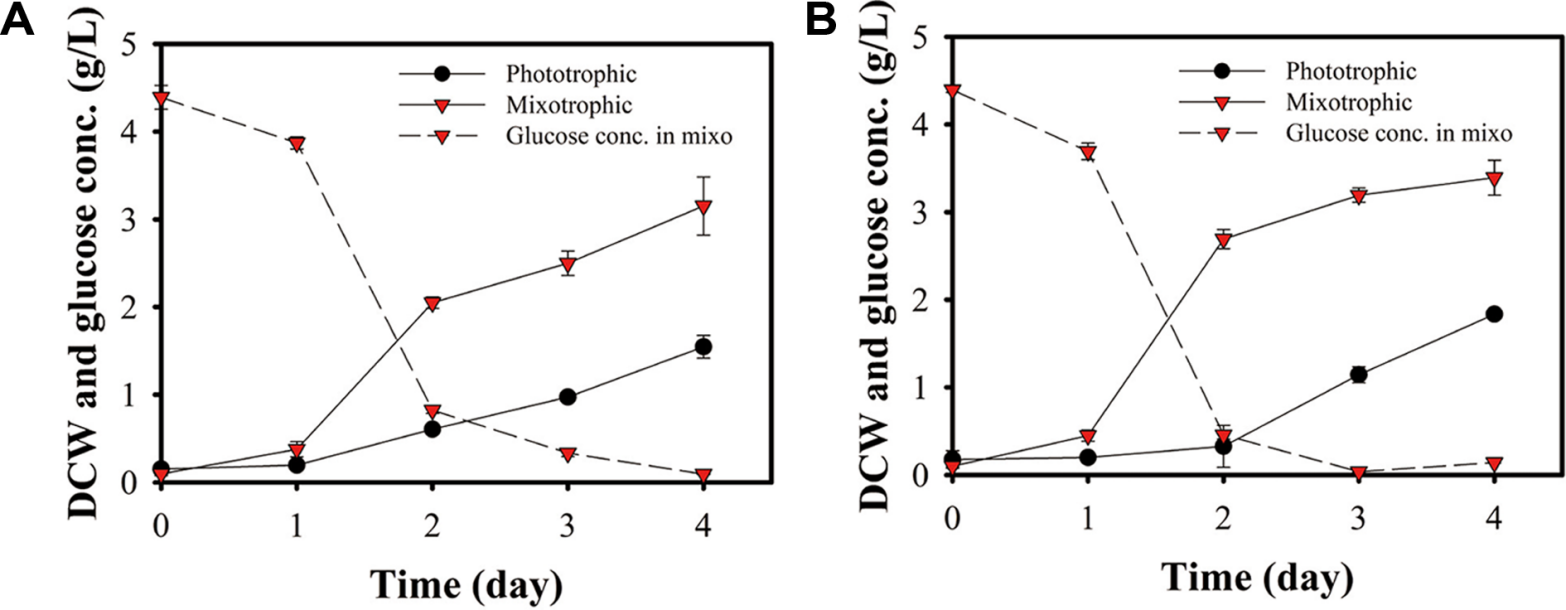
Profiles of cell growth under photo and mixotrophic conditions and glucose consumption. (**A**) *Chlorella* sp. ABC001. (**B**) *Chlorella* sp. HS2. Error bars indicate mean ± standard deviation (*n* = 3)

To induce different cell dividing property, two different culture conditions were applied. The first condition was regular photoautotrophic condition, utilizing CO_2_, light and nutrients from BG11 medium. While the other condition was mixotrophic, supplemented with additional glucose. Cell growth under both conditions were examined as shown in Fig. 1. For both strains (*Chlorella* sp. ABC001 and *Chlorella* sp. HS2), mixotrophic growth using glucose supplementation resulted in a higher growth rate and biomass yield throughout the entire cultivation period. Under photoautotrophic condition, *Chlorella* sp. ABC001 and *Chlorella* sp. HS2 reached biomass yields of 1.56 and 1.84 g/l, respectively, by day 4. However, under mixotrophic conditions, these values increased to 3.16 and 3.39 g/l, respectively by day 4. This enhanced growth in mixotrophic cultures was supported by the active consumption of supplied glucose (5 g/l).

In mixotrophic conditions, both *Chlorella* sp. ABC001 and HS2 exhibited a one-day lag phase. The highest glucose consumption rates (3.5 and 3.3 g glucose consumption/l/day, respectively) and the highest biomass increases (1.5 and 2.1 g biomass increase/l/day, respectively) were observed between day 1 and 2. After this period, growth rates declined to 0.55 and 0.35 g biomass/l/day, respectively, between day 2 and 4, which were similar to or lower than those observed in phototrophic conditions (0.47 and 0.73 g biomass/l/day, respectively). Although, residual glucose (0.32 g/l) remained in the medium, the lower growth of *Chlorella* sp. HS2 in mixotrophic conditions compared to phototrophic conditions suggests that glucose uptake may have suppressed photosynthesis [24]. This repression effect on photosynthesis has been reported to be species-dependent [25, 26]. In contrast, *Chlorella* sp. ABC001, exhibited a more moderate photosynthesis repression effect, despite having slightly higher residual glucose (0.72 g/l), and maintained a higher growth rate compared to the phototrophic condition.

### Different Profiles of Cell Size between Phototrophic and Mixotrophic Conditions

**Fig. 2:**
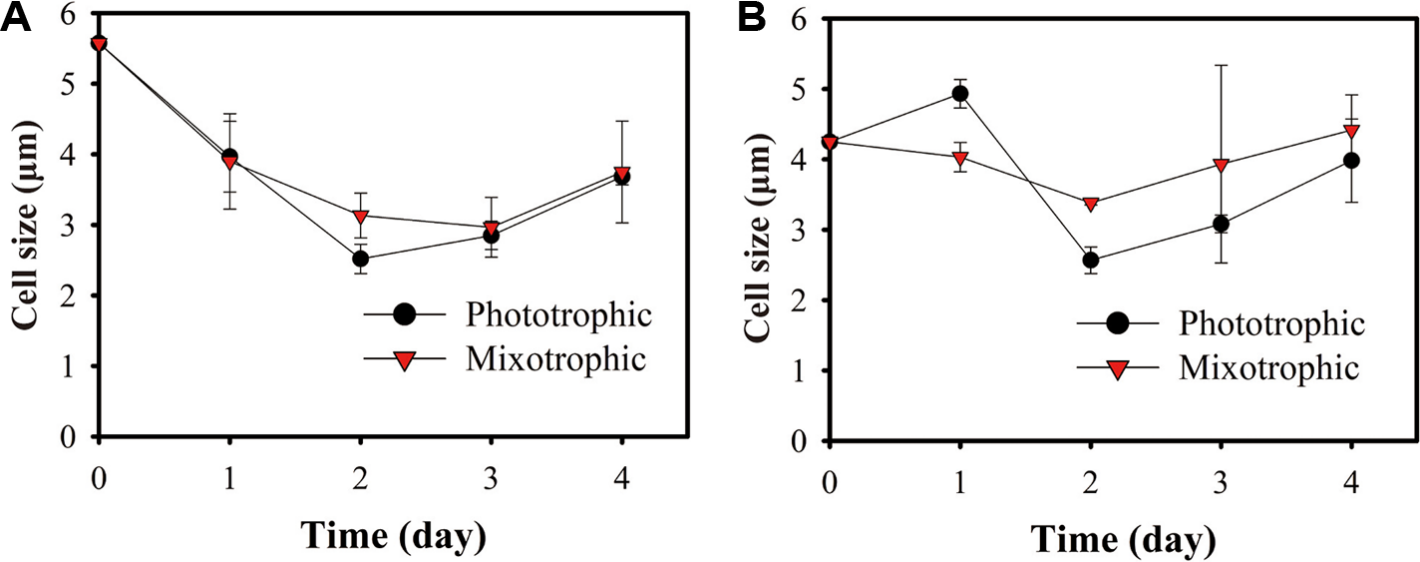
Changes in cell size under phototrophic and mixotrophic conditions. (**A**) *Chlorella* sp. ABC001. (**B**) *Chlorella* sp. HS2. Error bars indicate mean ± standard deviation (n ≥ 2000).

Cell size changes in *Chlorella* sp. ABC001 and *Chlorella* sp. HS2 under both phototrophic and mixotrophic conditions were examined as Fig. 2. For both strains, cell sizes tended to decrease until a certain time point (day 2) and then increased again. In the case of *Chlorella* sp. ABC001, both phototrophic and mixotrophic conditions started with an initial cell size of 5.57 mm, which decreased to 2.51 mm (phototrophic at day 2) and 3.13 mm (mixotrophic at day 2) (Fig. 2A). By day 4, the cell size increased to 3.68 mm (photoautotrophic) and 3.75 mm (mixotrophic). *Chlorella* sp. HS2 started with an initial size of 4.25 mm under both conditions (Fig. 2B). Under phototrophic conditions, the cell size increased slightly to 4.93 mm at day 1 but then decreased to 2.56 mm by day 2. In contrast, under mixotrophic conditions, the cell size decreased linearly to 3.38 mm by day 2. Subsequently, both conditions exhibited an increase in cell size by day 4, reaching 3.98 mm (photoautotrophic) and 4.41 mm (mixotrophic). The sharp decrease in cell size observed in both strains on day 2 is likely due to rapid cell division. It is well established that cell growth can occur through two mechanisms: Cell enlargement and an increase in cell number through cell division. In most cases, these processes are balanced, with cell enlargement occurring during interdivision periods, and fission taking place during division periods. It has been reported that rapid cell division without enough interdivision period, results in smaller daughter cells [27]. When cells encounter unfavorable condition, they tend to accumulate nutrient and delay division until the changes in the condition [28]. Conversely, when cells are exposed to favorable condition, then cells focus on the cell division and sometimes it leads unusually frequent cell division events [28]. In this experiment, both strains have faced favorable conditions with the introduction of fresh medium, making cell division the dominant response under all conditions. However, the reason for rapid cell division may differ between the conditions. In mixotrophic conditions, this rapid division could be correlated with the active consumption of glucose and an increase in dry cell weight until day 2. In contrast, under the phototrophic condition, *Chlorella* sp. ABC001 initiated growth after lag phase, whereas *Chlorella* sp. HS2 was still in the lag phage. After this period of rapid cell division, which resulted in reduced cell size, cells gradually increased in size under both conditions. In the case of *Chlorella* sp. ABC001, the cells were enlarged up to 3.68 mm (phototrophic condition) and 3.75 mm (mixotrophic condition) by day 4 and in the case of *Chlorella* sp. HS2, the cells were getting back to their initial cell size, reaching 3.98 mm (phototrophic condition) and 4.41 mm (mixotrophic condition). For this increase, several possible reasons could be suggested. One is that cells may have initially adapted to the new favorable conditions, which subsequently became less optimal for sustained growth. Also, rapid cell proliferation could have led to a shift in culture conditions, favoring cellular enlargement over further proliferation, as larger cells are capable of retaining greater amounts of nutrients, allowing them to sustain metabolic activities for longer durations [29]. These two reasons could be applied to both phototrophic and mixotrophic conditions. Furthermore, for mixotrophic conditions, rapid cell growth was initially supported by the availability of glucose, however, after the depletion of glucose, cells had to turning back to normal phototrophic growth, which contributed to the recovery of cell size for ordinary cell division. Thus, with this data, it was found that cell division dynamics was continuously changed during the linear growth phase by internal transitions of culture conditions including cellular or environmental factors.

### Changes in Ratio between DCW and OD at 750 nm

**Fig. 3:**
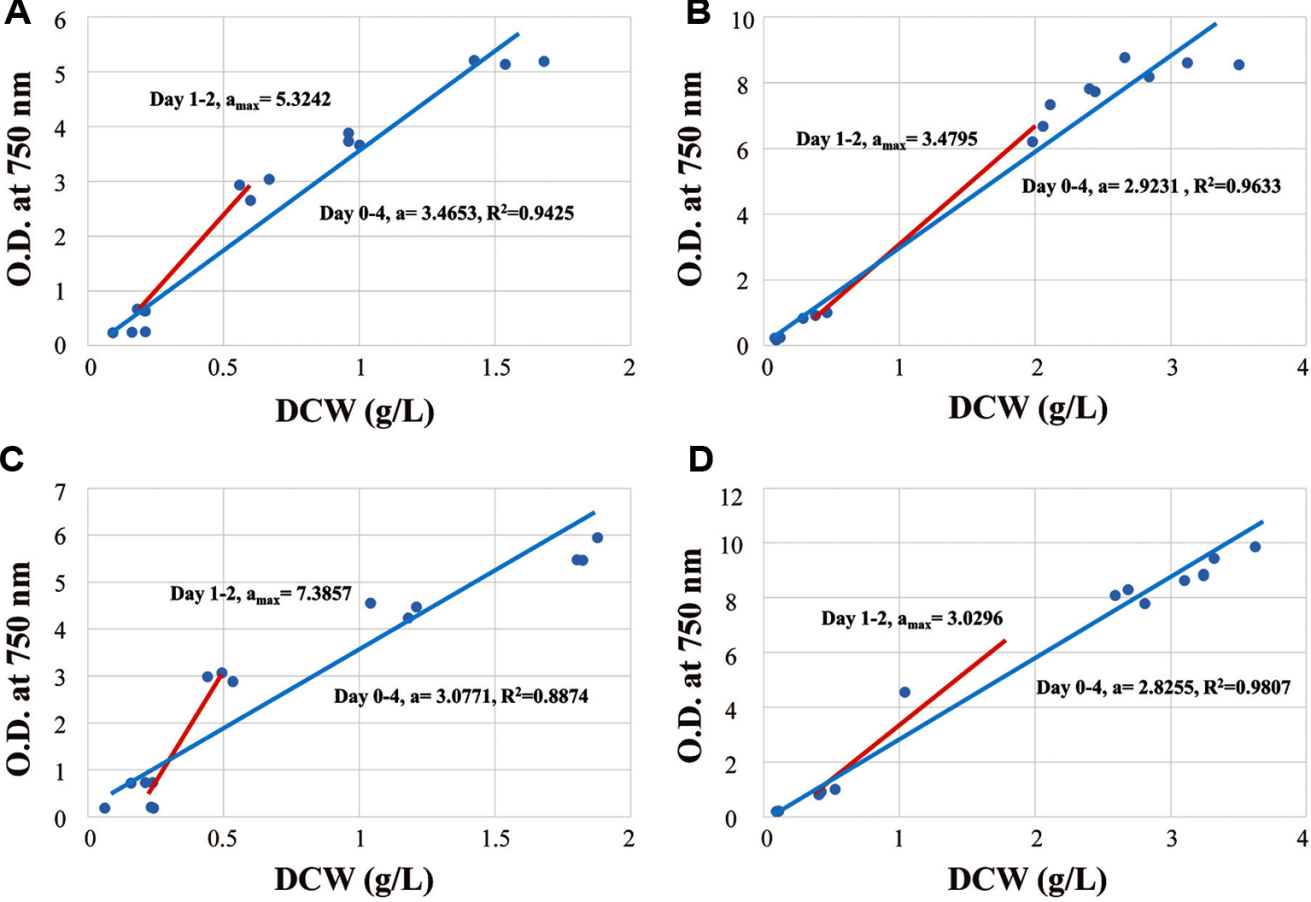
Conversions between DCW and Optical density at 750 nm. *Chlorella* sp. ABC001 under phototrophic condition (**A**) and mixotrophic condition (**B**). *Chlorella* sp. HS2 under phototrophic condition (**C**) and mixotrophic condition (**D**).

For the measurement of cell growth in microbiology, the relationship between OD and cell growth (DCW or cell number) was broadly used based on the assumption that there are no significant changes in cell morphology. However, as observed in the previous section, cell size changed during growth, thus, an examination of its effect on the relationship between OD and cell growth is required. In order to investigate this, DCW and OD at 750 nm were plotted as Fig. 3. As referred, lots of researchers have used OD for quick and easy cell growth measurement and correlate it with DCW for more precise assessment. The correlation between OD at 750 nm and DCW is typically expressed as linear function, y = ax + b where ‘a’ is a slope and ‘b’ is a constant. If the cell size changes during cultivation, the slope of linear relationship between OD and DCW will also change. For *Chlorella* sp. ABC001, the phototrophic condition showed an overall slope of 3.4653 with a maximum slope of 5.3242 between day 1 and 2. The mixotrophic condition had an overall slope of 2.9231 and the maximum slope of 3.4795 between day 1 and 2. Considering that ABC 001 cells under both conditions exhibited their minimum cell size on day 2, these maximum slope values could be correlated with the smallest cell size. In DCW vs. OD graphs, the slope can give insight into cell size. Assuming cells are spherical, OD at 750 nm (a turbidity-based measurement) is proportional to cell surface area, which is second order function of cell radius [30]. In contrast, DCW is determined by cellular density multiplied by cell volume, which is a third-order function of the cell radius. Thus, the slope of DCW vs. OD graph is proportional to the reciprocal of the cell radius. Therefore, when cell size reaches its minimum, the slope has the maximum value during cultivation. This explains why the maximum slope (5.3242) under phototrophic conditions exceeds that (3.4795) under mixotrophic condition, aligning with the smaller cell size (2.52 mm for phototropic vs. 3.13 mm for mixotrophic conditions). A similar pattern occurred with *Chlorella* sp. HS2. The highest slopes for phototrophic and mixotrophic conditions were 7.3857 and 3.0296, respectively both observed between day 1 and 2. The overall slope were 3.0771 (phototrophic) and 2.8255 (mixotrophic). Correspondingly, the smallest cell sizes occurred on day 2, measuring 2.8 mm (phototrophic) and 3.8 mm (mixotrophic). As expected, the phototrophic condition, with smaller cells, exhibited a steeper slope than the mixotrophic condition. In this way, the changes in the slope of DCW vs. OD graph can serve as an indicator of cell size variation driven by cell division dynamics. 

Morphological changes during cultivation have been studied in the other microbes, including yeast [31, 32], fungi [33, 34], and microalgae [35, 36]. Due to various research objectives (*e.g.*, enhancing target project yield, observing diverse morphologies, or analyzing the effect of culture conditions on cell differentiation) and differing measurement techniques (*e.g.* microscopy, laser particle size analysis, cytometry), their findings have been applied differently. In contrast to these prior studies, this research focuses on refining the basic method of OD-based growth estimation to enhance its reliability and precision.

### Changes in Chlorophyll Content

**Fig. 4:**
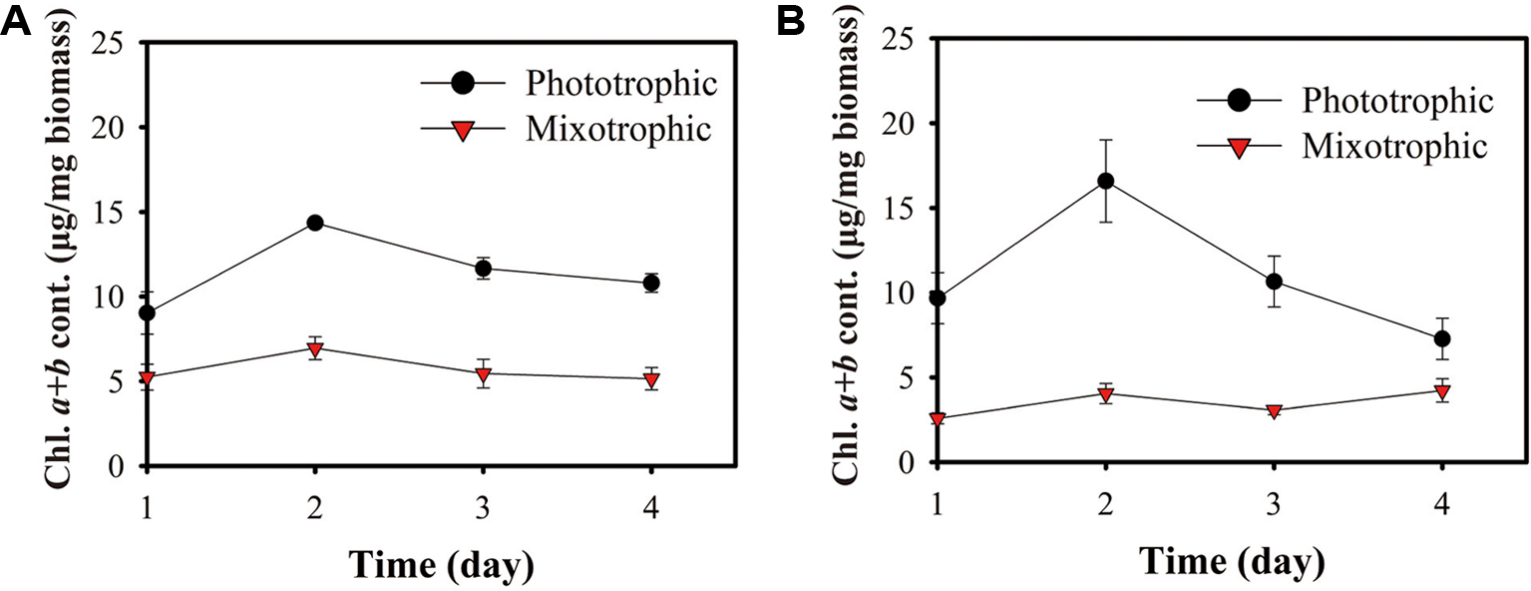
Changes in the chlorophyll content during the cultivation. (**A**) *Chlorella* sp. ABC001 under phototrophic and mixotrophic conditions. (**B**) *Chlorella* sp. HS2 under phototrophic and mixotrophic conditions. Error bars indicate mean ± standard deviation (*n* = 3)

The previous section demonstrated that different culture conditions resulted in varied growth performances, influencing cell division characteristics and cellular growth patterns. In general, the content of target product in algal biomass also varies during cultivation. Therefore, real-time monitoring of target products, cell division properties, and their relationship through the growth period is critical for optimizing production process. It has been reported that cell division properties are closely related to cellular metabolism, which determines the yield of target metabolites [27]. Therefore, in this section, we analyzed the chlorophyll content, a major product with antioxidant properties. First of all, under phototrophic conditions, the chlorophyll *a* + *b* content in *Chlorella* sp. ABC001 increased from 9.02 mg/mg biomass to peak of 14.5 mg/mg biomass on day 2, before decreasing to 10.79 mg/mg biomass on day 4 (Fig. 4A). A similar trend was observed under mixotrophic conditions, where chlorophyll *a* + *b* increased slightly from 5.24 mg/mg biomass to 6.95 mg/mg biomass on day 2 and then decreased to 5.14 mg/mg biomass on day 4. Compared to phototrophic conditions (maximum increase of 5.32 mg/mg biomass), the change in the chlorophyll content under mixotrophic conditions was minimal (less than 1.80 mg/mg biomass). *Chlorella* sp. HS2 showed a similar profile as Fig. 4B. Under phototrophic conditions, chlorophyll a+b content increased from 9.66 mg/mg biomass to 16.58 mg/mg biomass on day 2, then decreased to 7.27 mg/mg biomass on day 4. Under mixotrophic conditions, no significant variation was observed, with chlorophyll a+b content ranged from 2.58 to 4.22 mg/mg biomass. In this experiment, phototrophic conditions consistently resulted in higher chlorophyll content than mixotrophic conditions. As discussed earlier, glucose consumption under mixotrophic conditions suppresses photosynthesis by reducing the expression level of photosynthetic apparatus, including photosynthetic antenna complexes composed of various photosynthetic pigments [24]. The initial differentiation in the chlorophyll content (day 1) between phototrophic and mixotrophic conditions could be resulted from this photosynthetic repression. Once glucose was depleted, cells under mixotrophic conditions could not recover their chlorophyll content. However, under phototrophic conditions, cells rapidly produced chlorophyll to maximize their photosynthetic capacity until day 2, followed by a gradual decline in chlorophyll content. It has been reported that synthesized light-harvesting antennas generally have larger light harvesting capacity than the cells require for photosynthesis [37]. Considering that cell growth under mixotrophic cultivation proceeds effectively, the decrease in chlorophyll content cannot be attributed to nutrient limitation [38]. For a more detailed examination of the causes behind the decrease in chlorophyll content, further studies are required to explore the balance between higher photosynthetic efficiency and competition among the cells.

This study indicates that chlorophyll content is determined by culture conditions and is influenced by cellular division properties and the environmental context experienced by the cells. Changes in culture conditions modify cellular division patterns, resulting in differences in daughter cell size and even the concentration of cellular components. Considering that microalgae can be cultivated under phototrophic, mixotrophic or heterotrophic conditions, the productivity of target products can be vary significantly depending on the conditions and cultivation strategies used. Therefore, in order to maximize target product yields from microalgae biomass, it is crucial to select appropriate culture conditions. Simultaneously continuous monitoring and optimization of culture parameters, including cell division property and environmental conditions, are essential for process

## References

[1] Kang NK, Kim M, Baek K, Chang YK, Ort DR, Jin YS (2022). Photoautotrophic organic acid production: glycolic acid production by microalgal cultivation. Chem. Eng. J..

[2] Somers MD, Chen P, Clippinger J, Cruce JR, Davis R, Lammers PJ (2021). Techno-economic and life-cycle assessment of fuel production from mixotrophic Galdieria sulphuraria microalgae on hydrolysate. Algal Res..

[3] Ruiz J, Olivieri G, de Vree J, Bosma R, Willems P, Reith JH (2016). Towards industrial products from microalgae. Energy Environ. Sci..

[4] Park W-K, Yoo G, Moon M, Kim CW, Choi Y-E, Yang J-W (2013). Phytohormone supplementation significantly increases growth of *Chlamydomonas reinhardtii* cultivated for biodiesel production. Appl. Biochem. Biotechnol..

[5] Amaro HM, Guedes AC, Malcata FX (2011). Advances and perspectives in using microalgae to produce biodiesel. Appl. Energ..

[6] Skjånes K, Rebours C, Lindblad P (2013). Potential for green microalgae to produce hydrogen, pharmaceuticals and other high value products in a combined process. Crit. Rev. Biotechnol..

[7] Thiviyanathan VA, Ker PJ, Hoon Tang SG, Amin EPP, Yee W, Hannan MA (2024). Microalgae biomass and biomolecule quantification: optical techniques, challenges and prospects. Renew. Sustain. Energy Rev..

[8] Dupré C, Burrows HD, Campos MG, Delattre C, Encarnação T, Fauchon M, *et al*. 2020. Microalgal Biomass of Industrial Interest: Methods of Characterization, pp. 537-639. *In* Nzihou A (ed.), *Handbook on Characterization of Biomass, Biowaste and Related Byproducts*, Ed. Springer International Publishing, Cham.

[9] Guillard RRL, Sieracki MS. 2005. *Counting cells in cultures with the light microscope*, Algal cultureing techniques pp. 239-252. Acedemic Press, Cambridge.

[10] Sarrafzadeh MH, La H-J, Seo S-H, Asgharnejad H, Oh H-M (2015). Evaluation of various techniques for microalgal biomass quantification. J. Biotechnol..

[11] Moheimani NR, Borowitzka MA, Isdepsky A, Sing SF. 2013. Standard Methods for Measuring Growth of Algae and Their Composition, pp. 265-284. *In* Borowitzka MA, Moheimani NR (eds.), *Algae for Biofuels and Energy*, Ed. Springer Netherlands, Dordrecht.

[12] Daneshvar E, Sik Ok Y, Tavakoli S, Sarkar B, Shaheen SM, Hong H (2021). Insights into upstream processing of microalgae: a review. Bioresour. Technol..

[13] Beal J, Farny NG, Haddock-Angelli T, Selvarajah V, Baldwin GS, Buckley-Taylor R (2020). Robust estimation of bacterial cell count from optical density. Commun. Biol..

[14] Kirst H, Formighieri C, Melis A (2014). Maximizing photosynthetic efficiency and culture productivity in cyanobacteria upon minimizing the phycobilisome light-harvesting antenna size. Biochim. Biophysic. Acta - Bioenerg..

[15] Chioccioli M, Hankamer B, Ross IL (2014). Flow cytometry pulse width data enables rapid and sensitive estimation of biomass dry weight in the microalgae *Chlamydomonas reinhardtii* and *Chlorella vulgaris*. PLoS One.

[16] Nielsen SL, Hansen BW (2019). Evaluation of the robustness of optical density as a tool for estimation of biomass in microalgal cultivation: the effects of growth conditions and physiological state. Aquac. Res..

[17] Arbones B, Figueiras FG, Zapata M (1996). Determination of phytoplankton absorption coefficient in natural seawater samples: evidence of a unique equation to correct the pathlength amplification on glass fiber filters. Mar. Ecol. Prog. Ser..

[18] Myers JA, Curtis BS, Curtis WR (2013). Improving accuracy of cell and chromophore concentration measurements using optical density. BMC Biophys..

[19] Mittag M. 2001. *Circadian rhythms* in microalgae, pp. 213-247. *International Review of Cytology*, Ed. Academic Press. 10.1016/s0074-7696(01)06023-5 11407761

[20] de Winter L, Schepers LW, Cuaresma M, Barbosa MJ, Martens DE, Wijffels RH (2014). *Circadian rhythms* in the cell cycle and biomass composition of *Neochloris oleoabundans* under nitrogen limitation. J. Biotechnol..

[21] Cross FR, Umen JG (2015). The *Chlamydomonas* cell cycle. Plant J..

[22] Chan LL, Zhong X, Qiu J, Li PY, Lin B (2011). Cellometer vision as an alternative to flow cytometry for cell cycle analysis, mitochondrial potential, and immunophenotyping. Cytom. Part A..

[23] Ritchie RJ (2006). Consistent sets of spectrophotometric chlorophyll equations for acetone, methanol and ethanol solvents. Photosynth. Res..

[24] Oesterhelt C, Schmalzlin E, Schmitt JM, Lokstein H (2007). Regulation of photosynthesis in the unicellular acidophilic red alga *Galdieria sulphuraria*. Plant J..

[25] Wilken S, Schuurmans JM, Matthijs HC (2014). Do mixotrophs grow as photoheterotrophs? Photophysiological acclimation of the chrysophyte *Ochromonas danica* after feeding. New Phytol..

[26] Abiusi F, Wijffels RH, Janssen M (2020). Doubling of microalgae productivity by oxygen balanced mixotrophy. ACS Sustain. Chem. Eng..

[27] Bišová K, Zachleder V (2014). Cell-cycle regulation in green algae dividing by multiple fission. J. Exp. Bot..

[28] Bauer A, Minceva M (2021). Examination of photo-, mixo-, and heterotrophic cultivation conditions on *Haematococcus pluvialis* cyst cell germination. Appl. Sci..

[29] Sánchez-Alvarez EL, González-Ledezma G, Bolaños Prats JA, Stephano-Hornedo JL, Hildebrand M (2017). Evaluating *Marinichlorella kaistiae* KAS603 cell size variation, growth and TAG accumulation resulting from rapid adaptation to highly diverse trophic and salinity cultivation regimes. Algal Res..

[30] Gregory J (1998). Turbidity and beyond. Filtr. Sep..

[31] Aon JC, Tecson RC, Loladze V (2018). *Saccharomyces cerevisiae* morphological changes and cytokinesis arrest elicited by hypoxia during scale-up for production of therapeutic recombinant proteins. Microb. Cell Fact..

[32] Ohnuki S, Enomoto K, Yoshimoto H, Ohya Y (2014). Dynamic changes in brewing yeast cells in culture revealed by statistical analyses of yeast morphological data. J. Biosci. Bioeng..

[33] El Enshasy HA (2022). Fungal morphology: a challenge in bioprocess engineering industries for product development. Curr. Opin. Chem. Eng..

[34] Müller H, Barthel L, Schmideder S, Schütze T, Meyer V, Briesen H (2022). From spores to fungal pellets: a new high-throughput image analysis highlights the structural development of *Aspergillus niger*. Biotechnol. Bioeng..

[35] Yan P, Guo J-s, Zhang P, Xiao Y, Li Z, Zhang S-q (2021). The role of morphological changes in algae adaptation to nutrient stress at the single-cell level. Sci. Total Environ..

[36] Ahmad S, Kothari R, Shankarayan R, Tyagi VV (2019). Temperature dependent morphological changes on algal growth and cell surface with dairy industry wastewater: an experimental investigation. 3 Biotech..

[37] Friedland N, Negi S, Vinogradova-Shah T, Wu G, Ma L, Flynn S (2019). Fine-tuning the photosynthetic light harvesting apparatus for improved photosynthetic efficiency and biomass yield. Sci. Rep..

[38] Mandalam RK, Palsson B (1998). Elemental balancing of biomass and medium composition enhances growth capacity in highdensity *Chlorella vulgaris* cultures. Biotechnol. Bioeng..

